# Environmental Stress Responses of *DnaJA1*, *DnaJB12* and *DnaJC8* in *Apis cerana cerana*

**DOI:** 10.3389/fgene.2018.00445

**Published:** 2018-10-08

**Authors:** Guilin Li, Hang Zhao, Xuemei Zhang, Yanming Zhang, Huayu Zhao, Xinxin Yang, Xingqi Guo, Baohua Xu

**Affiliations:** ^1^State Key Laboratory of Crop Biology, College of Life Sciences, Shandong Agricultural University, Tai’an, China; ^2^College of Animal Science and Technology, Shandong Agricultural University, Tai’an, China

**Keywords:** *Apis cerana cerana*, environmental stresses, DnaJ, expression analysis, RNA interference, functional analysis

## Abstract

DnaJ, also known as Hsp40, plays important roles in maintaining the normal physiological state of an organism under stress conditions by mediating essential processes, such as protein synthesis, degradation, folding and metabolism. However, the exact functions of most DnaJ members are not fully understood in insects. Here, we identified three genes, *AccDnaJA1*, *AccDnaJB12*, and *AccDnaJC8*, in *Apis cerana cerana* and explored their connection with the environmental stress response. Quantitative real-time PCR results showed that the mRNA levels of *AccDnaJA1*, *AccDnaJB12*, and *AccDnaJC8* were all induced under cold, UV, H_2_O_2_ and different pesticides treatment. The expression patterns of *AccDnaJB12* and *AccDnaJC8* were upregulated by CdCl_2_ and HgCl_2_ stress, while the transcriptional levels of *AccDnaJA1* were downregulated by CdCl_2_ and HgCl_2_ stress. Western blot findings further indicated that AccDnaJB12 protein levels were increased by some stress conditions. Knockdown of each of these three genes downregulated the transcriptional patterns of several stress response-related genes at different levels. Functional analysis further demonstrated that the resistance of *A. cerana cerana* to lambda-cyhalothrin stress was reduced with knockdown of *AccDnaJA1*, *AccDnaJB12*, or *AccDnaJC8*, indicating that these three genes may be involved in the tolerance to this pesticide. Taken together, these findings indicate that *AccDnaJA1*, *AccDnaJB12*, and *AccDnaJC8* may play pivotal roles in the stress response by facilitating honeybee survival under some adverse circumstances. To our knowledge, this is the first report that reveals the roles of DnaJ family proteins under different adverse circumstances in *A. cerana cerana*.

## Introduction

Heat shock proteins (HSPs) are molecular chaperones well known for their function in mediating inappropriate aggregation, folding, misfolding and unfolding of proteins (Kim et al., 2013). These tasks are essential for organisms not only under normal conditions but also under diverse environmental stresses, and the need for HSPs is increased under non-optimal environmental conditions (Rinehart et al., 2007). Based on their molecular weight in kDa, HSPs are divided into six families including small HSP (sHsp), Hsp40, Hsp60, Hsp70, Hsp90, and Hsp100 (Sørensen et al., 2010; Requena et al., 2015). In recent years, the characteristics of each family of HSPs, including Hsp40, had been gradually revealed.

Hsp40 was first identified to stimulate DnaK (an Hsp70 homolog in bacteria) ATPase activity to assist with replication of host cell λ phage DNA (Yochem et al., 1978; Liberek et al., 1988). It is often named DnaJ for the presence of a J domain, which consists of approximately 70 amino acids, in its protein structure (Jiang et al., 2007). In addition, according to the difference in the number and type of domain structure, DnaJ proteins are divided into three classes, DnaJA, DnaJB, and DnaJC. DnaJA contains a J domain, variable C-terminal domain, cysteine-rich region and glycine/phenylalanine-rich region. DnaJB has all the domains presented above except for the cysteine-rich region, while DnaJC only contains a J domain (Vos et al., 2008). Previous studies had indicated that DnaJ usually cooperated with DnaK to participate in diverse cellular biological processes, such as protein export, protein folding, DNA replication and stress responses by relying on ATP hydrolysis (Craig et al., 2006). Fully investigating the role of DnaJ will contribute to understanding the functional mechanism of DnaJ/DnaK in different biological processes under stress conditions.

Recently, some studies revealed the biological function of DnaJ under stressful environmental conditions. For example, overexpression of *DnaJA1* in human pancreatic cancer cells represses the stress response capacity of c-Jun, possibly through activation of the DnaK protein, triggering a reduction in cell survival (Stark et al., 2014). Overexpression of *DnaJ*/*DnaK* increases the freeze tolerance capacity of *Escherichia coli*, probably by restraining irreversible protein denaturation (Chow and Tung, 1998). Under acid stress for 0.25 and 1 h, the mRNA levels of *DnaJ* are increased 2.1- and 35.7-fold, respectively, in *Alicyclobacillus acidoterrestris* (Jiao et al., 2012). ERdj4, an endoplasmic reticulum DnaJ homolog, can stimulate BiP ATPase activity and is significantly upregulated at both the mRNA and protein levels following endoplasmic reticulum stress in mice (Shen et al., 2002). ERdj5 is also a DnaJ protein in the endoplasmic reticulum, contains a thioredoxin and DnaJ domain and is expressed in response to endoplasmic reticulum stress in humans (Cunnea et al., 2003). Despite the progress achieved in DnaJ research, the functions of many members of DnaJ have not been uncovered in many species, including honeybees.

As one type of Asian honeybee, *Apis cerana cerana* brings substantial ecosystem services to various terrestrial plants and produces tremendous economic benefits for people. Recently, however, due to the influence of diverse environmental stresses, the honeybee population size had obviously decreased in some regions (Potts et al., 2010; Kulhanek et al., 2017; Li et al., 2018b). Exploring the stress response mechanism at gene expression regulation level will be beneficial to the stress-resistance biology research of honeybees. Furthermore, a recent study reported that insect HSPs played important roles during stress (King and MacRae, 2015), and our previous studies also demonstrated that four sHsp genes (*AccHsp22.6*, *AccHsp23.0*, *AccHsp24.2*, and *AccHsp27.6*) were involved in the stress response in *A. cerana cerana* (Liu et al., 2012; Liu Z. et al., 2014; Zhang et al., 2014). These results indicate that HSPs may play key roles in defense against environmental stress in honeybees.

To gain insight into the function of the DnaJ family of HSPs in *A. cerana cerana*, we identified three DnaJ genes, named *AccDnaJA1*, *AccDnaJB12*, and *AccDnaJC8*, in this study and investigated their response to environmental stress. The results of quantitative real-time PCR (qRT-PCR) analysis revealed that the expression levels of *AccDnaJA1*, *AccDnaJB12*, and *AccDnaJC8* were induced under many stress conditions. Western blot analysis further proved that AccDnaJB12 was upregulated by some stress factors at the protein level. An RNA interference (RNAi) experiment indicated that knockdown of *AccDnaJA1*, *AccDnaJB12*, and *AccDnaJC8* repressed the transcriptional profiles of several stress response-related genes and decreased the survival ability of *A. cerana cerana* under lambda-cyhalothrin stress. Taken together, our findings shed light on the stress response mechanisms of *AccDnaJA1*, *AccDnaJB12*, and *AccDnaJC8* in honeybees.

## Materials and Methods

### Prediction of the Possible *Cis*-Acting Elements Mapping to the *AccDnaJA1*, *AccDnaJB12*, and *AccDnaJC8* 5′-Flanking Regions

The online software TFBIND^1^ and MatInspector database^2^ were selected to predict the probable *cis*-acting elements in the 5′-flanking regions of *AccDnaJA1* (Gene ID: 107996076), *AccDnaJB12* (Gene ID: 108000381), and *AccDnaJC8* (Gene ID: 107998514) (Tsunoda and Takagi, 1999).

### Honeybee Rearing

The honeybee colonies used for the experiment were raised at the College of Animal Science and Technology, Shandong Agricultural University (Tai’an, China). The 15-day-old *A. cerana cerana* foragers were distinguished by marking the newly emerged honeybees with pigment 15 days in advance. The honeybee colonies were normally managed every day by our beekeeper to maintain a strong and healthy colony.

### Diverse Environmental Stress Exposure

Recent studies had shown that honeybees were often subjected to multiple environmental stresses, and the foragers (approximately 2–3 weeks old) usually had more opportunity for exposure to adverse circumstances (Potts et al., 2010; Even et al., 2012; Farooqui, 2014; Robertson et al., 2014). In this study, we selected 15-day-old foragers to use for the expression analysis and functional analysis of *AccDnaJA1* (GenBank accession no. XP 016909435.1), *AccDnaJB12* (GenBank accession no. XP 016916168.1) and *AccDnaJC8* (GenBank accession no. XP 016913324.1). The 15-day-old foragers were collected from the strong and healthy *A. cerana cerana* colonies and divided into 13 groups. Among these groups, 11 groups were randomly exposed to one of the following treatments including 4, 14, and 24°C, ultraviolet radiation (UV), CdCl_2_, HgCl_2_, lambda-cyhalothrin, emamectin benzoate, spirodiclofen, avermectin, and paraquat.

For the 4, 14, and 24°C treatments, 3 groups of honeybees were reared in incubators set at 4, 14, and 24°C, respectively, with 70% relative humidity in the dark. For UV treatment, the honeybees were directly irradiated with 30 mJ/cm^2^ UV using a desk type UV lamp in an incubator (33°C, 70% relative humidity and 24 h dark). With regard to CdCl_2_ and HgCl_2_ treatment, honeybees in two groups were injected with 1 μL CdCl_2_ (2 g/mL) or HgCl_2_ (2 g/mL), respectively, between the second abdominal segment (A2) and third abdominal segment (A3), which was defined according to the online database^3^, using a microsyringe. During injected, our operators grasped the two pairs of wings of honeybees by one hand to immobilize them. For pesticide treatment, five groups of honeybees were maintained in incubators that were sprayed with lambda-cyhalothrin (0.05 mg/mL), emamectin benzoate (2 mg/mL), spirodiclofen (2 mg/mL), avermectin (0.05 mg/mL), or paraquat (2 mg/mL) in advance, separately, and kept at 33°C with 70% relative humidity in the dark. The remaining two groups were both used as control groups. Among them, one group was kept untreated and used as the control group for the 4, 14, and 24°C, ultraviolet radiation (UV), lambda-cyhalothrin, emamectin benzoate, spirodiclofen, avermectin, and paraquat treatments. The other group that injected with 1 μL sterilized water between the second abdominal segment (A2) and third abdominal segment (A3) was the control group for the CdCl_2_ and HgCl_2_ treatments. All the groups were fed fresh pollen dough and a 30% sucrose solution. Then, the healthy honeybees in each group were sampled at specific times (**Supplementary Table S1**) and kept in an ultralow temperature freezer (Panasonic, Japan) until use.

### RNA Extraction, cDNA Synthesis, and qRT-PCR Analyses

Total RNA from whole honeybees was extracted using RNAiso Plus (TaKaRa, Dalian, China) according to the manufacturer’s instructions. The first strand of cDNA was acquired using a HiScript^®^ II Q RT SuperMix for qPCR (+gDNA wiper) Kit (Vazyme, Nanjing, China) following the supplier’s instructions. The qRT-PCR analyses were carried out using SYBR^®^ *Premix Ex Taq*^TM^ (Tli RNaseH Plus) (TaKaRa, Dalian, China) and a CFX96^TM^ Real-Time System. The efficiency values of all pairs of primers used for qRT-PCR were all within 90–110%. Each pair of qRT-PCR primers had a single peak, and their correlation coefficients (*R*^2^) were approaching 1. The specific sequences of qRT-PCR primers are presented in **Supplementary Table S2**. The β-actin gene (GenBank accession no. HM640276.1) was used as a standard to normalize other gene expression levels as it is stably expressed in honeybees (Lourenço et al., 2008; Yao et al., 2014). For each treatment, at least three biological replicates were performed. The 2^-ΔΔ^*^C^*^T^ method and CFX Manager software (version 3.1) were used to analyze the relative levels of gene expression.

### Preparation of Anti-AccDnaJA1 and Anti-AccDnaJB12

The coding region of AccDnaJA11 and part of coding region of AccDnaJB12 were, respectively, fused into the prokaryotic expression vector pET-30a (+) (Novagen, Darmstadt, Germany). Then these two recombinant vectors were, respectively, transformed into Transetta (DE3) chemically competent cells (TransGen Biotech, Beijing, China), and the cell were induced with 0.3 mM isopropyl-1-thio-bgalactopyranoside at 37°C for 10 h during logarithmic growth phase. The 12% SDS-PAGE was used to separate the recombinant proteins (AccDnaJA11 and AccDnaJB12), which was used to inject into the white mouse (Taibang, Tai’an, China) to further acquire anti-AccDnaJA11 and anti-AccDnaJB12, and the remaining operation methods were performed as described as Meng et al. (2010) and Li et al. (2016a).

### Extraction of Protein and Western Blot Analysis

Total protein from whole honeybee was extracted using a tissue protein extraction kit (ComWin Biotech, Beijing, China). After denaturation, total protein was separated by 12% Tris-glycine gels and electrotransferred onto PVDF membranes (ComWin Biotech, Beijing, China) through a semidry method. Anti-AccDnaJA11 and anti-AccDnaJB12 antibodies [1:500 (v/v) dilution] were used as the primary antibodies, and a horseradish peroxidase-conjugated goat anti-mouse antibody (1:2000 (v/v) dilution, Dingguo Changsheng Biotechnology, Beijing, China) was served as the secondary antibody. The binding reaction was detected using a SageCapture^TM^ Biological imager (Beijing Sage Creation Science Co., Ltd., Beijing, China) and the Immobilon^TM^ Western Chemiluminescent HRP Substrate (Millipore Corporation, Billerica, MA, United States).

### The Synthesis of Double-Stranded RNA (dsRNA)

Primers specific to partial *AccDnaJA1*, *AccDnaJB12*, and *AccDnaJC8* coding regions were constructed with a T7 promoter sequence at each primer 5′ end. The primer sequences can be found in **Supplementary Table S2**. *EasyTaq*^®^ DNA Polymerase (TransGen Biotech, Beijing, China) and a DNA Extraction Kit (Solarbio, Beijing, China) were used to perform PCR amplifications and purify the PCR products following standard procedures, respectively. The PCR amplification procedures were presented in **Supplementary Table S3**. A T7 RiboMAX^TM^ Express RNAi System (Promega, Madison, United States) and the purified PCR products were used to produce large quantities of dsRNA for *AccDnaJA1*, *AccDnaJB12*, and *AccDnaJC8* according to the manufacturer’s instructions. As a control, we also produced dsRNA for green fluorescent protein (GFP) (GenBank accession no. U87974) (Wang et al., 2012).

### Knockdown of *AccDnaJA1*, *AccDnaJB12*, and *AccDnaJC8* in *Apis cerana cerana*

The 15-day-old foragers were divided into four groups (*n* = 30). Groups 1–3 were fed 7 μg dsRNA AccDnaJA1, dsRNA AccDnaJB12 and dsRNA AccDnaJC8, respectively, and as a control, group 4 was fed 7 μg dsRNA GFP. Then, the four groups were raised in an incubator (34°C, 70% relative humidity and 24 h darkness) and fed fresh pollen dough and a 30% sucrose solution. The healthy honeybees were sampled for verification of RNAi efficiency 2 days after ingestion of dsRNA.

### The Transcriptional Levels of Some Stress Response-Related Gene After Knockdown of *AccDnaJA1*, *AccDnaJB12*, and *AccDnaJC8*

After RNAi of *AccDnaJA1*, *AccDnaJB12*, and *AccDnaJC8*, qRT-PCR was used to detect the mRNA levels of *AccANT*, *AccCDK5r*, *AccCPR24*, *AccERR*, *AccTP1*, *AccCDK5*, *AccCYP336A1*, *AccSp10*, *AccRBM11*, *AccAK*, *AccMTNR1A*, and *AccAIF3*. These genes were proved to be involved in the stress response in our previous studies (Chu et al., 2013; Liu X. et al., 2014; Wang et al., 2014; Chen et al., 2015; Li et al., 2016b, 2018a; Zhang et al., 2016; Zhou et al., 2016; Zhu et al., 2016; Gao et al., 2017; Zhao et al., 2018).

### Lambda-Cyhalothrin Tolerance Ability Experiment Under RNAi Treatment

The 15-day-old foragers were divided into four groups (*n* = 50) fed dsRNA AccDnaJA1, dsRNA AccDnaJB12, dsRNA AccDnaJC8, or dsRNA GFP (7 μg/individual), separately. Two days later, the four groups were all exposed to lambda-cyhalothrin stress as described in the “Diverse Environmental Stress Exposure” section, and then the survival rate of each group was recorded every 0.5 h. At least three biological replicates were performed.

### Statistical Analysis

Duncan’s multiple range tests of Statistical Analysis System software (version 9.1) were used to determine the differences between multiple groups. The mean ± SE from three independent experiments was denoted by the standard error bars. Different letters above the error bars were used to present the significant differences. Significant difference and no significant difference between various groups were indicated by the different letter and the same letter, respectively. If there was difference between two groups and the difference was not significant, the overlapped letters were used to indicate statistical significance. ^∗^*P* < 0.05 and ^∗∗^*P* < 0.01 were determined by the Student’s *t*-test.

## Results

### Expression of Recombinant AccDnaJA1 and AccDnaJB12 Protein

As a histidine-tag fusion protein, AccDnaJA1 was overexpressed in *E. coli* Transetta (DE3). An SDS-PAGE analysis indicated that this recombinant protein was expressed successfully in Transetta and had obvious stripes between 44.3 and 66.4 kDa, which contained 44.828 kDa AccDnaJA1 and approximately 7 kDa cleavable N- and C-terminal His-tags (**Supplementary Figure S1A**). The induced overexpression of pET-30a(+) vector Transetta and uninduced overexpression of pET-30a(+)-AccDnaJA1 were used as control.

In regard to the recombinant AccDnaJB12 protein, when its whole coding region was fused into prokaryotic expression vector pET-30a (+), the Transetta (DE3) could not express this recombinant protein. The signal peptide and transmembrane domain usually influence the express ability of *E. coli* to target heterologous protein, and we found that the protein of AccDnaJB12 had a transmembrane domain near the sit of 250 amino acid. Therefore, we selected the amino acid sequence from 1 to 210 to fuse to prokaryotic expression vector pET-30a (+). The truncated AccDnaJB12 protein is 24.068 kDa, when added to approximately 7 kDa cleavable N- and C-terminal His-tags of the vector pET-30a (+), the recombinant AccDnaJB12 protein is approximately 31 kDa. Consistent with this predicted result, the truncated recombinant AccDnaJB12 protein was successfully expressed (**Supplementary Figure S1B**).

### Partial Predictable *Cis*-Acting Elements in the 5′-Flanking Region of *AccDnaJA1*, *AccDnaJB12*, and *AccDnaJC8*

To preliminarily explore the functions of *AccDnaJA1*, *AccDnaJB12*, and *AccDnaJC8* with regard to the stress response, partial 5′-flanking regions of *AccDnaJA1*, *AccDnaJB12*, and *AccDnaJC8* were chosen to predict possible *cis*-acting elements using TFBIND. We found many putative binding sites bound by stress response-related transcription factors, such as p53, NF-κB, CREB, and AP-1 (Wan et al., 2012; Bagul et al., 2015; Charni-Natan et al., 2018; Chen et al., 2018; Fujimura and Usuki, 2018; Russo et al., 2018), in the 5′-flanking regions of *AccDnaJA1*, *AccDnaJB12*, and *AccDnaJC8*, and a partial list of them is shown in **Table 1**.

**Table 1:** Predictable stress response-related transcription factors and their binding sites in the 5′ flanking regions of *AccDnaJA1*, *AccDnaJB12*, and *AccDnaJC8*.

Target gene	Transcription factor	Putative position	Possible binding site
*AccDnaJA1*	NF-κB	36 (+)	AATGGAATTTC CAC
		37 (–)	ATGGAATTTC CACC
	CREB	131 (+)	TTACGACA
		131 (–)	TTACGACA
	AP-1	188 (+)	AATGATTCTGA
		220 (+)	GTTGAATAAAA
		398 (+)	ATCTGTCACTTA GTCGTAA
		820 (–)	TTTATTCAA
		1174 (+)	TTTAATAAA
	p53	236 (+)	ATGCATGCTT
		651 (+)	AAACGAGTTT
		1344 (–)	TAACTTGTAA
*AccDnaJB12*	AP-1	11 (+)	TATGAATTAAT
		21 (–)	TTTATTAAA
		251 (+)	TGTGACAAAAA
		442 (–)	AATTATTCATT
		586 (–)	ATTATTAAT
	p53	178 (+)	ATGCTAGTTA
		327 (–)	AAACACATCT
		630 (–)	AGACATTTTG
	NF-κB	611 (–)	AAATAAATTTCATT
	CREB	667 (–)	AGATATGTCAGC
		826 (–)	TGCTCCGTCATT
*AccDnaJC8*	AP-1	239 (–)	CTAAGACAT
		252 (+)	TTTAATAAA
		577 (–)	TTGGATTCATT
		787 (+)	AATGATGAAGA
		1207 (+)	TTGATTCAG
	p53	275 (–)	GAATAAGTCA
		1062 (+)	ATATTTGCTT
	CREB	1109 (–)	AAATTCGTCAAA
		1438 (+)	CGTGACGTATGT


### The Expression Levels of *AccDnaJA1*, *AccDnaJB12*, and *AccDnaJC8* and Their Proteins Under Cold, Heavy Metal and UV Stresses

**FIGURE 1:**
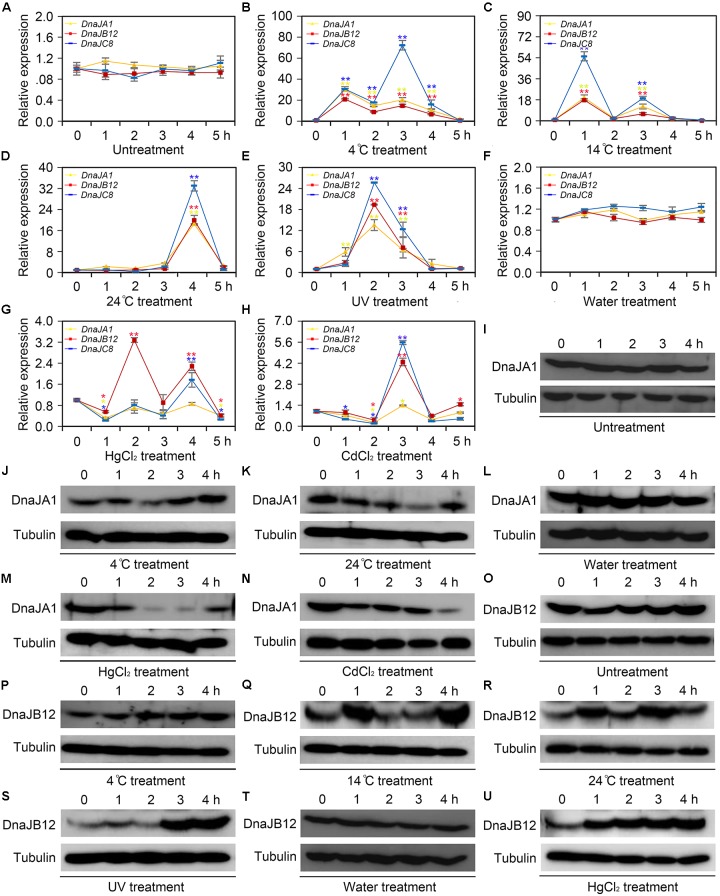
Transcription and translation levels analysis of *AccDnaJA1*, *AccDnaJB12*, and *AccDnaJC8* under cold, UV and heavy metal stress. **(A–H)** qRT-PCR was performed to reveal the mRNA levels of *AccDnaJA1*, *AccDnaJB12*, and *AccDnaJC8*. **(A)** qRT-PCR results of the control group of 4°C **(B)**, 14°C **(C)**, 24°C **(D)**, and UV **(E)**. **(F)** qRT-PCR results of the control group of HgCl_2_ **(G)** and CdCl_2_ **(H)**. Mean ± SE of three replicates of six individuals each. ^∗^*P* < 0.05, ^∗∗^*P* < 0.01 by the Student’s *t*-test. Yellow, red, and blue asterisk above the error bars indicate significant differences of *AccDnaJA1*, *AccDnaJB12*, and *AccDnaJC8*, respectively. The β-actin gene was used as an internal control. **(I–U)** Western blot analysis of AccDnaJA1 and AccDnaJB12. Equivalent quality of total protein from *Apis cerana cerana* was loaded for each sample. The target proteins were immunoblotted with anti-AccDnaJA1 or anti-AccDnaJB12. Tubulin was used as a control. **(I)** The control group of AccDnaJA1 under 4°C **(J)** and 24°C **(K)** treatment. **(L)** The control group of AccDnaJA1 under HgCl_2_ **(M)** and CdCl_2_ **(N)** treatment. **(O)** Western blot results of the control group of AccDnaJB12 under 4°C **(P)**, 4°C **(Q)**, and 24°C **(R)** treatment. **(T)** Western blot results of the control group of AccDnaJB12 under HgCl_2_ **(U)** treatment.

To determine whether *AccDnaJA1*, *AccDnaJB12*, and *AccDnaJC8* responded to cold (4, 14, and 24°C), heavy metal (CdCl_2_ and HgCl_2_) and UV stress at the transcriptional level, qRT-PCR was carried out, and the β-actin gene was used as an internal control. As shown in **Figure 1A**, the mRNA levels of *AccDnaJA1*, *AccDnaJB12*, and *AccDnaJC8* were little changed under normal condition within 5 h, while they were all upregulated and reached their highest levels at 1, 1, and 3 h, respectively, under 4°C stress (**Figure 1B**). At 14°C, *AccDnaJA1*, *AccDnaJB12*, and *AccDnaJC8* were all increased and reached their maximum levels at 1 h (**Figure 1C**). Under stress of 24°C, all three genes reached a peak at 4 h (**Figure 1D**). Under UV stress, *AccDnaJA1*, *AccDnaJB12*, and *AccDnaJC8* were all upregulated with a peak at 2 h, and then all were gradually downregulated (**Figure 1E**). The expression levels of *AccDnaJA1*, *AccDnaJB12*, and *AccDnaJC8* were nearly not changed when honeybees were injected sterilized water (**Figure 1F**). However, when honeybees were injected with HgCl_2_, *AccDnaJB12* was induced and reached the highest transcript level at 2 h, and *AccDnaJA1* and *AccDnaJC8* was repressed in response to HgCl_2_ stress (**Figure 1G**). With CdCl_2_ treatment, *AccDnaJA1* was downregulated, while *AccDnaJB12* and *AccDnaJC8* were both upregulated at 3 h (**Figure 1H**). These results suggest that *AccDnaJA1*, *AccDnaJB12*, and *AccDnaJC8* can respond to the above six stress conditions to a certain degree at transcriptional level.

To investigate the protein levels of AccDnaJA1 under some stress conditions, western blot was carried out. The protein profiles of AccDnaJA1 were not altered under normal condition (**Figure 1I**), while were increased under 4°C stress (**Figure 1J**). The protein levels of AccDnaJA1 were downregulated under 24°C stress (**Figure 1K**), and were not obviously regulated under 14°C stress (**Supplementary Figure S2**). The expression levels of AccDnaJA1 were not changed when honeybees were injected sterilized water, while were inhibited under both HgCl_2_ and CdCl_2_ stress (**Figures 3L–N**), which was consistent with the results from qRT-PCR. These findings suggest that AccDnaJA1 may play a role in various stress conditions at different degrees.

Then, we used western blot to examine the protein levels of AccDnaJB12 under some stress conditions. The protein levels of AccDnaJB12 were nearly not altered under normal condition (**Figure 1O**). The expression levels of AccDnaJB12 were increased under treatment at 4, 14, and 24°C (**Figures 1P–R**). UV stress caused an increase in AccDnaJB12 levels at 3.0 and 4.0 h (**Figure 1S**). When injected sterilized water, the protein levels of AccDnaJB12 were not changed (**Figure 1T**), while injected with HgCl_2_ triggered a continuous upregulation of the protein levels of AccDnaJB12 from 1.0 to 4.0 h (**Figure 1U**). These findings indicate that AccDnaJB12 may play a pivotal role in some environmental stress response.

### The Relative mRNA or Protein Levels of *AccDnaJA1*, *AccDnaJB12*, and *AccDnaJC8* in Response to Different Pesticide Stresses

**FIGURE 2:**
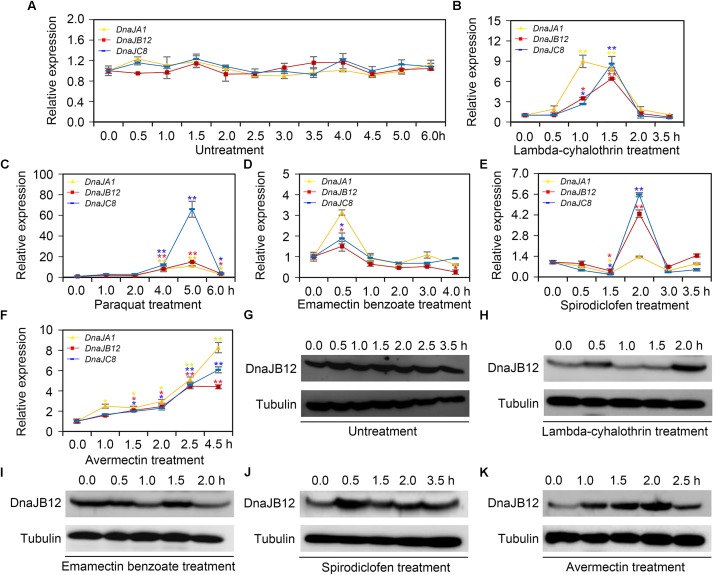
The relative expression of *AccDnaJA1*, *AccDnaJB12*, and *AccDnaJC8* under several pesticide stresses. **(A–F)** The mRNA levels of *AccDnaJA1*, *AccDnaJB12*, and *AccDnaJC8* under normal condition **(A)**, and lambda-cyhalothrin **(B)**, paraquat **(C)**, emamectin benzoate **(D)**, spirodiclofen **(E)**, and avermectin **(F)** treatment. The expression patterns of *AccDnaJA1*, *AccDnaJB12*, and *AccDnaJC8* were normalized to that of the β-actin gene. The data are shown as the mean ± SE from at least three replicates of six individuals each. Yellow, red, and blue asterisk above the error bars indicate significant differences of *AccDnaJA1*, *AccDnaJB12*, and *AccDnaJC8*, respectively (^∗^*P* < 0.05, ^∗∗^*P* < 0.01 by the Student’s *t*-test). **(G–K)** The protein levels of AccDnaJB12 under normal **(G)**, lambda-cyhalothrin **(H)**, emamectin benzoate **(I)**, spirodiclofen **(J)**, and avermectin **(K)** conditions. The control was tubulin.

When collecting water, pollen and nectar, honeybees are often suffered from pesticides used in agriculture (Goulson et al., 2015). To explore the mRNA levels of *AccDnaJA1*, *AccDnaJB12*, and *AccDnaJC8* compared to the β-actin gene under various pesticide stresses, qRT-PCR was performed. The mRNA levels of *AccDnaJA1*, *AccDnaJB12*, and *AccDnaJC8* were not obviously changed under normal condition (**Figure 2A**). In response to lambda-cyhalothrin treatment, *AccDnaJA1*, *AccDnaJB12*, and *AccDnaJC8* were all upregulated with the highest mRNA levels at 1.0, 1.5 and 1.5 h, respectively (**Figure 2B**). Both *AccDnaJA1*, and *AccDnaJC8* were increased from 1 to 5 h under paraquat stress, and *AccDnaJB12* was also upregulated from 1 to 5 h except at 2 h when honeybees were subjected to paraquat stress (**Figure 2C**). Compared to the effects of the above two pesticide stresses, emamectin benzoate had only a slightly influence on the transcriptional levels of *AccDnaJA1*, *AccDnaJB12*, and *AccDnaJC8* (**Figure 2D**). As presented in **Figure 2E**, all three genes were induced with a peak at 2 h by spirodiclofen stress. Under avermectin stress, *AccDnaJA1*, *AccDnaJB12*, and *AccDnaJC8* were all upregulated continuously from 1.0 to 4.5 h (**Figure 2F**).

Then we used western blot to detect the protein levels of AccDnaJB12 under some pesticide stresses. As shown in **Figure 2G**, the translation levels of AccDnaJB12 were not significantly regulated under normal condition from 0 h to 3.5 h, while were increased at 0.5 and 2.0 h under lambda-cyhalothrin stress (**Figure 2H**). When *A. cerana cerana* was exposed to emamectin benzoate stress, the AccDnaJB12 protein levels were repressed at 1.0 and 2.0 h (**Figure 2I**). Though the AccDnaJB12 protein levels were induced in response to both the spirodiclofen and avermectin treatments, it seemed that the upregulation varied by stress condition as spirodiclofen triggered maximum protein expression at 0.5 h and avermectin caused maximum protein expression at 2.0 h (**Figures 2J,K**).

### Investigation of the Efficiency of dsRNA-Induced *AccDnaJA1*, *AccDnaJB12*, and *AccDnaJC8* Silencing

**FIGURE 3:**
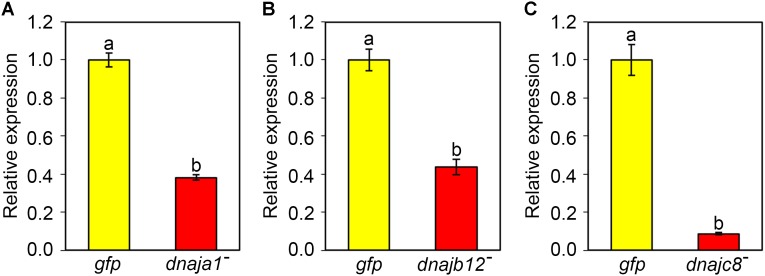
Efficiency of RNAi on the transcriptional levels of *AccDnaJA1* **(A)**, *AccDnaJB12* **(B)**, and *AccDnaJC8* **(C)**. For validation, total RNA was acquired from 15-day-old foragers after feeding with 7 μg dsRNA AccDnaJA1, dsRNA AccDnaJB12, dsRNA AccDnaJC8, or dsRNA GFP for 2 days. qRT-PCR was performed to examine the mRNA profiles of *AccDnaJA1*, *AccDnaJB12*, and *AccDnaJC8* normalized against β-actin. The abbreviations *dnaja1*^-^, *dnajb12*^-^, *dnajc8*^-^, and *gfp* indicate *AccDnaJA1* knockdown, *AccDnaJB12* knockdown, *AccDnaJC8* knockdown and *GFP* control, respectively. Mean ± SE of three replicates of six individuals each. Various letters above bars suggest significant differences between two groups (*p* < 0.01) based on Duncan’s multiple range tests.

qRT-PCR was used to verify the efficiency of RNAi-mediated gene silencing. After 2 days of feeding dsRNA AccDnaJA1, AccDnaJB12, or AccDnaJC8 to the honeybees, the transcriptional levels of *AccDnaJA1*, *AccDnaJB12*, and *AccDnaJC8* compared to that of the β-actin gene were tested. As presented in **Figure 3A**, *AccDnaJA* was successfully silenced compared to the expression level in the control group fed dsRNA GFP. *AccDnaJB12* and *AccDnaJC8* were also knocked down compared with the expression levels in the dsRNA GFP-fed group (**Figures 3B,C**).

### Expression Profiles of Other Stress Response Genes After Knockdown of *AccDnaJA1*, *AccDnaJB12*, and *AccDnaJC8*

**FIGURE 4:**
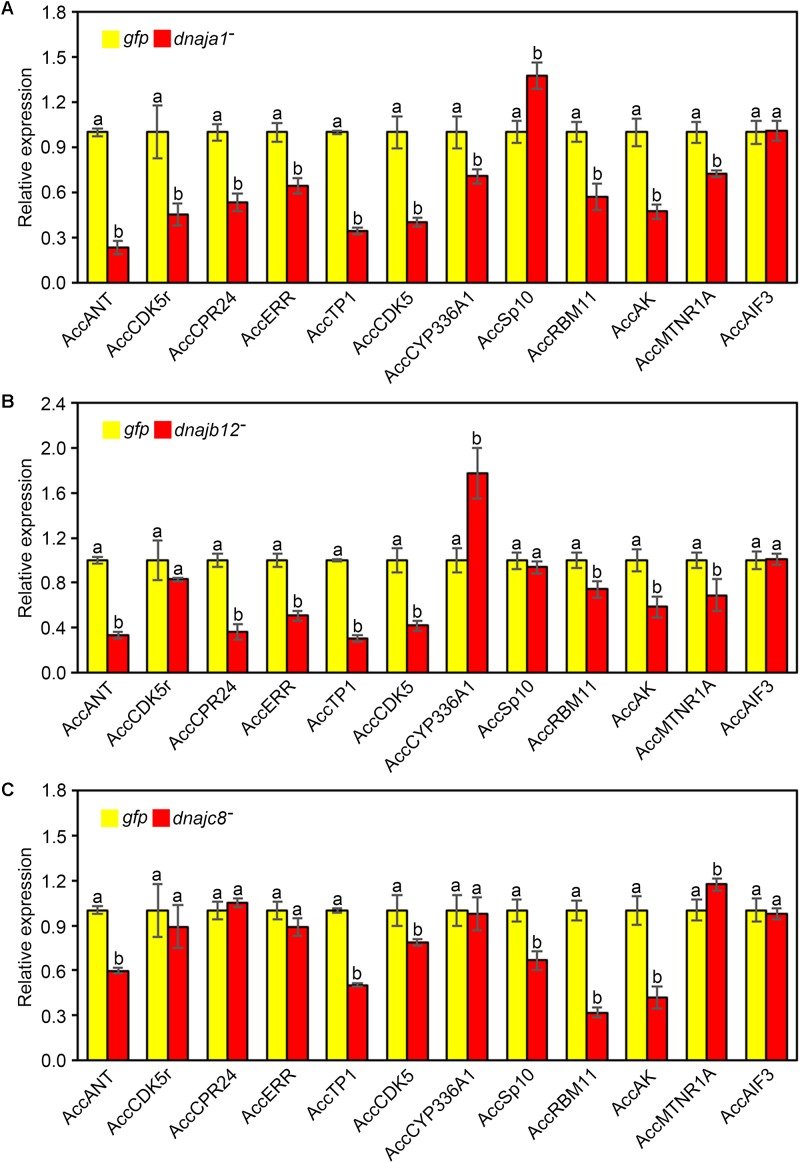
Effects of *AccDnaJA1*, *AccDnaJB12*, and *AccDnaJC8* knockdown on the other stress response genes. Relative mRNA levels of several stress response genes were investigated by qRT-PCR compared to that of the β-actin gene when silencing *AccDnaJA1* **(A)**, *AccDnaJB12* **(B)**, and *AccDnaJC8* **(C)**. *AccDnaJA1* knockdown, *AccDnaJB12* knockdown, *AccDnaJC8* knockdown, and *GFP* control were abbreviated as *dnaja1*^-^, *dnajb12*^-^, *dnajc8*^-^, and *gfp*, respectively. Mean ± SD are given (*n* = 3 with six individuals each). Significant differences between two groups were presented by various letters above the bars according to Duncan’s multiple range tests.

The effects of RNAi of *AccDnaJA1*, *AccDnaJB12*, and *AccDnaJC8* on the transcription levels of several stress response genes were investigated using qRT-PCR, and the β-actin gene was used as an internal control. As shown in **Figure 4A**, the mRNA levels of *AccANT*, *AccCDK5r*, *AccCPR24*, *AccERR*, *AccTP1*, *AccCDK5*, *AccCYP336A1*, *AccRBM11*, *AccAK*, and *AccMTNR1A* were inhibited when *AccDnaJA1* was knocked down, while the transcriptional levels of *AccSp10* were slightly upregulated. When *AccDnaJB12* was silenced, the expression levels of *AccANT*, *AccCPR24*, *AccERR*, *AccTP1*, *AccCDK5*, *AccRBM11*, *AccAK*, and *AccMTNR1A* were downregulated, and the transcriptional levels of *AccCYP336A1* were upregulated approximately 1.7-fold (**Figure 4B**). Knockdown of *AccDnaJC8* decreased the expression profiles of *AccANT*, *AccTP1*, *AccCDK5*, *AccSp10*, *AccRBM11*, and *AccAK* (**Figure 4C**). These findings indicate that the mRNA profiles of several stress response genes are influenced by knockdown of *AccDnaJA1*, *AccDnaJB12*, and *AccDnaJC8*, and *AccDnaJA1*, *AccDnaJB12*, and *AccDnaJC8* may play important roles in stress response.

### RNAi-Induced Silencing of *AccDnaJA1*, *AccDnaJB12*, or *AccDnaJC8* Decreased the Lambda-Cyhalothrin Tolerance of the Honeybee

**FIGURE 5:**
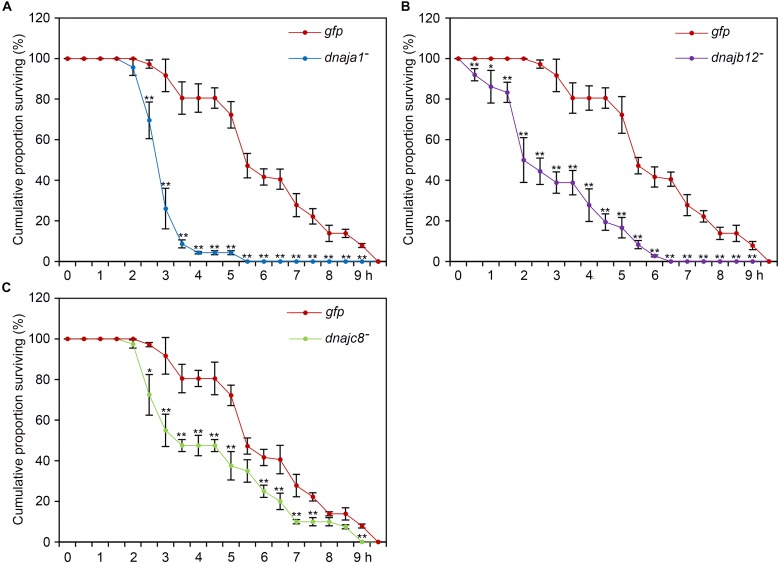
Lambda-cyhalothrin stress tolerance of *Apis cerana cerana* after RNAi *AccDnaJA1* **(A)**, *AccDnaJB12* **(B)**, and *AccDnaJC8* **(C)**. Survival rate of honeybees after a 10 h exposure to lambda-cyhalothrin when knockdown of *AccDnaJA1*, *AccDnaJB12*, and *AccDnaJC8* was recorded. The abbreviations *dnaja1*^-^, *dnajb12*^-^, *dnajc8*^-^, and *gfp* indicate *AccDnaJA1* RNAi, *AccDnaJB12* RNAi, *AccDnaJC8* RNAi, and GFP control, respectively. Mean ± SE of three replicates of 50 individuals each. The significant differences of *AccDnaJA1*, *AccDnaJB12*, and *AccDnaJC8* were indicated by asterisk, respectively (^∗^*P* < 0.05 and ^∗∗^*P* < 0.01 by the Student’s *t*-test).

To further demonstrate the role of *AccDnaJA1*, *AccDnaJB12*, and *AccDnaJC8* in the stress conditions, a functional analysis was performed using the RNAi experiment. After 2 days of feeding with dsRNA AccDnaJA1, AccDnaJB12, or AccDnaJC8, the honeybees were exposed to lambda-cyhalothrin stress, and we found that the removal of *AccDnaJA1*, *AccDnaJB12*, or *AccDnaJC8* decreased the survival ability of *A. cerana cerana* to a certain degree compared with the dsRNA GFP treatment group (**Figure 5**). These results suggest that *AccDnaJA1*, *AccDnaJB12*, and *AccDnaJC8* are involved in lambda-cyhalothrin tolerance in honeybee.

## Discussion

Heat shock proteins play roles as molecular chaperones in stress conditions by binding abnormally folded proteins, mediating the detrimental influence of misfolding and facilitating the return of these proteins to their native conformations at the time a favorable environment once again prevails (Feder and Hofmann, 1999; Rinehart et al., 2007). Though extensive studies have suggested that some members of the HSPs are generally expressed during periods of environmental stress, the expression regulation models of many DnaJ family genes are still unclear (Cheong et al., 2013; King and MacRae, 2015). In this study, we presented that the transcriptional levels of three DnaJ genes (*AccDnaJA1*, *AccDnaJB12*, and *AccDnaJC8*) were regulated under different environmental stresses, and western blot assays further supported our results. Furthermore, knockdown of *AccDnaJA1*, *AccDnaJB12*, or *AccDnaJC8* inhibited the expression of several stress response-related genes and increased the mortality rate of honeybees under lambda-cyhalothrin treatment. Our results thus reveal that *AccDnaJA1*, *AccDnaJB12*, and *AccDnaJC8* may play important roles in defense against adversity.

Accurate analysis of the role of a gene needs prediction of transcription factor binding ability to its regulatory regions. The 5′-flanking regions of *AccDnaJA1*, *AccDnaJB12*, and *AccDnaJC8* included many binding sites for NF-κB, CREB, AP-1, and p53 (**Table 1**), all of which are well-known transcription factors related to stress response. For example, the stress response pathway TIF-IA-NF-κB plays important roles in human colorectal tumors exposed to aspirin and chemopreventive agents (Chen et al., 2018). The activation of the p38 MAPK-CREB signaling pathway under MeHg-induced oxidative stress is conducive to neuronal cell death and cerebrocortical neuronal hyperactivity (Fujimura and Usuki, 2018). Fra1, a member of the transcription factor family of AP-1, is associated with metal treatment response in *Paracentrotus lividus* embryos (Russo et al., 2018). In HepG2 cells, p53 can change the different secretome of the liver and can influence whole organism homeostasis and liver function under various stress stimuli (Charni-Natan et al., 2018). Therefore, *AccDnaJA1*, *AccDnaJB12*, and *AccDnaJC8* may be involved in the stress response in *A. cerana cerana*.

Previous data had suggested that low temperature might cause chilling injury in insects induced by protein conformational changes and membrane phase transitions, and minor temperature changes triggering cold damage are possibly far less predictable (Ramlov, 2000; Sinclair et al., 2003). To cope with the injury from cold, insects use a series of strategies, including upregulated expression of HSPs (Rinehart et al., 2007). For example, *Drosophila melanogaster* *Hsp90*, *Hsp83*, *Hsp68*, *Hsp40*, *Hsp27*, *Hsp26*, *Hsp23*, and *Hsp22* are cold inducible, and knockdown of *Hsp23* and *Hsp22* by RNAi perturbs *D. melanogaster* recovery from low-temperature injury (Colinet et al., 2010; Colinet and Hoffmann, 2012). In our study, under cold stress, *AccDnaJA1*, *AccDnaJB12*, and *AccDnaJC8* were all increased at the transcriptional level or the protein level (**Figures 1B–D**). Honeybees usually congregate to acquire body heat to address cold stress at the behavioral level. The inducible expression of *AccDnaJA1*, *AccDnaJB12*, and *AccDnaJC8* may be a kind of decision to defend against cold stress at the gene expression regulation level.

Recent studies demonstrated that heavy metal and UV stress significantly influenced HSP expression and thereby affected the fitness and resistance of a natural population of organisms (Sørensen et al., 2010; Chen and Zhang, 2015). The majority of sHSPs present expression variation in response to multiple heavy metals in *Plutella xylostella* (Chen and Zhang, 2015). *Hsp70* displays particular sensitivity to zinc and iron exposure, while *Hsp60* shows the most sensitive response to manganese and cadmium treatments in *Litopenaeus vannamei* (Qian et al., 2012). The transcription levels of *Hsp27* are induced by UV stress in mouse skin tumors (Kiriyama et al., 2001). In addition, UV is likely to induce HSP expression, which may provide an adaptive response accompanied by increased exposure to UV radiation (Trautinger et al., 1996). In our study, *AccDnaJA1* was suppressed by both HgCl_2_ and CdCl_2_ treatment, while *AccDnaJB12* and *AccDnaJC8* were induced by HgCl_2_ and CdCl_2_ treatment (**Figures 1G,H**). The western blot experiment further proved our results (**Figures 1M,N**). The mRNA levels of *AccDnaJA1* and *AccDnaJC8* were upregulated by UV stress (**Figure 1E**), and both the transcription and translation of *AccDnaJB12* were increased under UV conditions (**Figures 1E,S**). These findings suggest that HSPs, at least *AccDnaJA1*, *AccDnaJB12*, and *AccDnaJC8*, are involved in heavy metal and UV stress.

Herbicides and insecticides are usually used in agriculture by humans to defend against ruderal or injurious insects. However, they also cause obvious damage to honeybees. Herbicides decrease honeybees’ food resource diversity, whereas insecticides directly kill honeybees by influencing their immunocompetence, behavior and antioxidant ability when interacting with different pathogens (Sanchez-Bayo et al., 2016; Li et al., 2018b). A previous study also proved that exposure to field-realistic doses of neonicotinoid pesticides decreased honeybee health in Canada’s corn-growing regions by using realistic experiments (Tsvetkov et al., 2017). Furthermore, recent studies reported that HSPs were involved in pesticide stress. The mRNA levels of *Hsp27* are significantly increased when *Chironomus riparius* is exposed to triclosan and bisphenol stress (Martinez-Paz et al., 2014). The expression profiles of 13 sHSPs are increased under chlorfenapyr and beta-cypermethrin stress in *P. xylostella* (Chen and Zhang, 2015). Our results indicated that the mRNA levels of *AccDnaJA1*, *AccDnaJB12*, and *AccDnaJC8* were all increased by pesticide stress (**Figures 2B–F**), and western blot results showed that the protein levels of AccDnaJB12 were upregulated under lambda-cyhalothrin, spirodiclofen, and avermectin stresses (**Figures 2H,J,K**). These results suggest that *AccDnaJA1*, *AccDnaJB12*, and *AccDnaJC8* may play an essential role in resisting pesticide stress in honeybees.

Notably, the transcriptional levels and translational levels of and *AccDnaJA1* *AccDnaJB12* were not exactly the same under some stress conditions (**Figures 1**, **2**). Differences in the transcriptional levels and translational levels of *invE* were also found by a previous study in *Shigella sonnei* (Mitobe et al., 2009). The following explanations may be considered for this difference. First, the transcription and translation of genes are regulated by different signaling pathways. The transcription of genes is mainly affected by core promoter and enhancer (Dikstein, 2011), and the translation of genes are governed by various factors, such as mRNA structure, RNA biding protein, 5′ or 3′ untranslated regions (Wang et al., 2015). For example, the interaction between HBV pregenomic RNA 5′ terminal redundancy and RNA-binding motif protein 24 inhibits the translation of core protein, while the interaction between RNA-binding motif protein 24 and the 3′ terminal redundancy increased HBV RNA stability (Yao et al., 2018). Second, multiple adverse circumstances can upregulate the mRNA levels of a gene but not enough to affect translation. Third, the protein can be degraded and accumulated in the body. For example, when the transcription of a gene is induced, the decreased protein expression levels may exist due to protein degradation.

RNAi is an effective tool for loss-of-function studies across eukaryotes, especially for organisms in which transgenosis is too hard to realize (Hannon, 2002; Fellmann and Lowe, 2014). Knockdown by RNAi also presents the importance of HSPs for the survival of insects in response to adverse circumstances (Rinehart et al., 2007; Fellmann and Lowe, 2014). To further demonstrate the connection of *AccDnaJA1*, *AccDnaJB12*, and *AccDnaJC8* with pesticide stress, RNAi was used. Functional analysis proved that separate knockdown of *AccDnaJA1*, *AccDnaJB12*, and *AccDnaJC8* decreased the survival rate of *A. cerana cerana* under lambda-cyhalothrin stress (**Figure 5**), suggesting that these three genes may protect honeybees from lambda-cyhalothrin stress to some extent. Furthermore, knockdown of *AccDnaJA1*, *AccDnaJB12*, or *AccDnaJC8* decreased the transcriptional levels of several stress response-related genes (**Figure 4**). These results further indicate that these three DnaJ genes play an essential role during the stress response for *A. cerana cerana*.

It has been indicated that the roles of individual proteins from the same HSP subfamily often differ in a variety of environmental conditions (King and MacRae, 2015). In this paper, the differential expression of *AccDnaJA1*, *AccDnaJB12*, or *AccDnaJC8* in different stressors may suggest their functional differences to some extent. The differences of specific molecular mechanisms of *AccDnaJA1*, *AccDnaJB12*, or *AccDnaJC8* in response to adverse circumstances are likely to contribute to their differential expression under stress conditions. In addition, these three genes were induced or repressed at different levels under multiple environmental stresses (**Figures 1**, **2**), indicating that their regulation roles in response various stressors might have distinction to a certain degree. Transgenic technology, RNA-seq and proteomics can be used for further investigating the reason of the differential expression of *AccDnaJA1*, *AccDnaJB12*, and *AccDnaJC8* in environmental stress in the future.

Collectively, our work identified three DnaJ genes (*AccDnaJA1*, *AccDnaJB12*, and *AccDnaJC8*) in *A. cerana cerana*. The expression levels of *AccDnaJA1*, *AccDnaJB12*, and *AccDnaJC8* in response to diverse environmental stresses indicate that these three genes may play key roles in stress-related defense mechanisms. Knockdown of these three genes repressed the mRNA levels of several stress response genes and increased the mortality rate of honeybees to a kind of pesticide (lambda-cyhalothrin) stress, which further demonstrated the roles of *AccDnaJA1*, *AccDnaJB12*, and *AccDnaJC8* in antistress circumstances. These results may be helpful for further unraveling the roles of the insect DnaJ family in stressful environmental conditions in future.

## Author Contributions

XG and BX planned and supervised the experiments. GL, HaZ, XZ, and YZ performed the experiments. GL, HaZ, HuZ, and XY analyzed the data. GL, BX, and XG wrote the paper. All authors approved the final version of the manuscript.

## Conflict of Interest Statement

The authors declare that the research was conducted in the absence of any commercial or financial relationships that could be construed as a potential conflict of interest.

## Footnotes

1 http://tfbind.hgc.jp/

2 http://www.cbrc.jp/research/db/TFSEARCH.html

3 http://honeybee.drawwing.org/book/externalanatomy

## Supplementary Material

FIGURE S1 The expression of the recombinant AccDnaJA1 or AccDnaJB12. Recombinant AccDnaJA1 **(A)** and AccDnaJB12 **(B)** were separated by 12% SDS-PAGE. Lanes 1: protein molecular weight marker; Lanes 2–3: overexpression of pET-30a(+) and uninduced overexpression of pET-30a(+)-target protein, respectively; Lanes 4–9: induced overexpression target protein. The sites of target genes were marked with a red box.

FIGURE S2 The protein levels of AccDnaJA1 under 14°C treatment. The target proteins were immunoblotted with anti-AccDnaJA1 and tubulin was used as a control.

TABLE S1 The collection times after treatment for the experimental groups.

TABLE S2 Primer sequences used in this study.

TABLE S3 PCR amplification procedures used in this study.
