# Supplementary figures and images for: Environmental Stress Responses of DnaJA1, DnaJB12 and DnaJC8 in Apis cerana cerana
Source: Front Genet. 2018 Oct 8;9:445. doi: 10.3389/fgene.2018.00445 (PMC6186841; doi:10.3389/fgene.2018.00445)

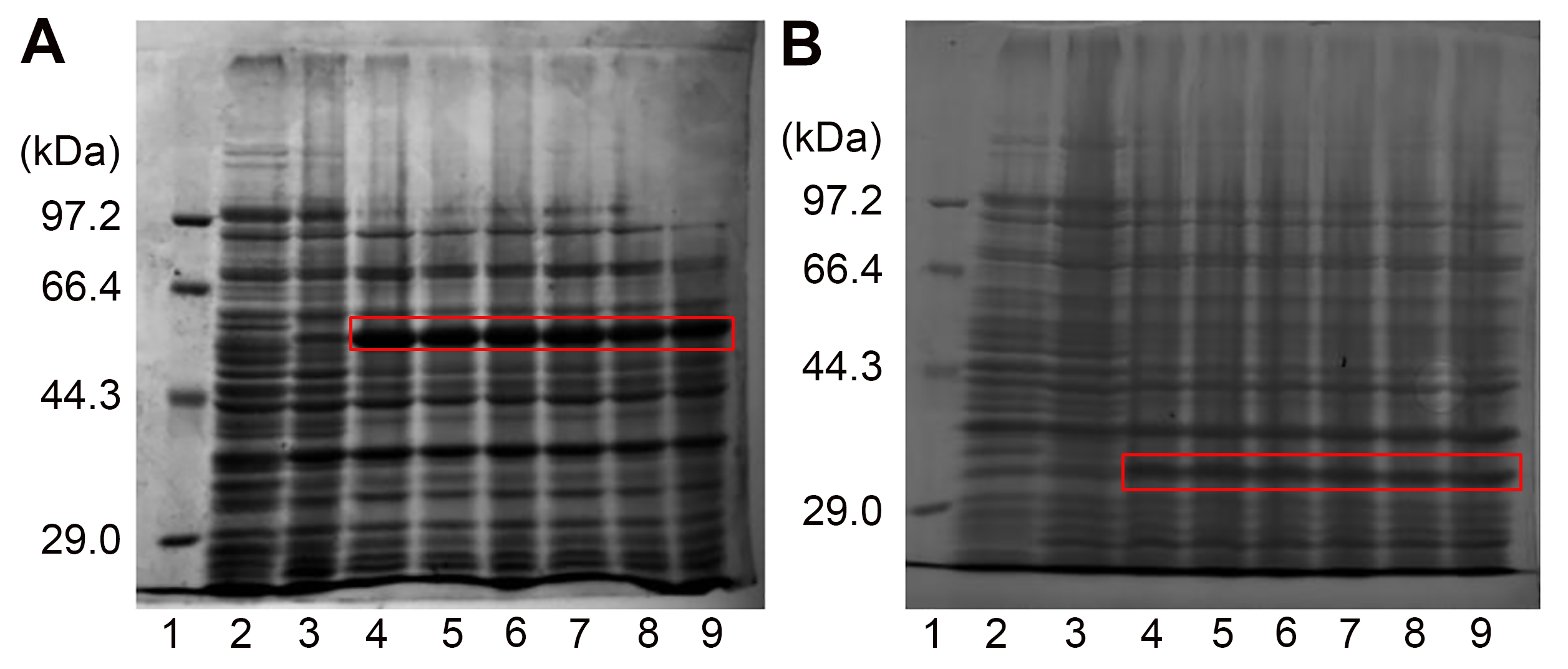

Supplement: FIGURE S1 — The expression of the recombinant AccDnaJA1 or AccDnaJB12. Recombinant AccDnaJA1 (A) and AccDnaJB12 (B) were separated by 12% SDS-PAGE. Lanes 1: protein molecular weight marker; Lanes 2–3: overexpression of pET-30a(+) and uninduced overexpression of pET-30a(+)-target protein, respectively; Lanes 4–9: induced overexpression target protein. The sites of target genes were marked with a red box. [file Image_1.TIF]

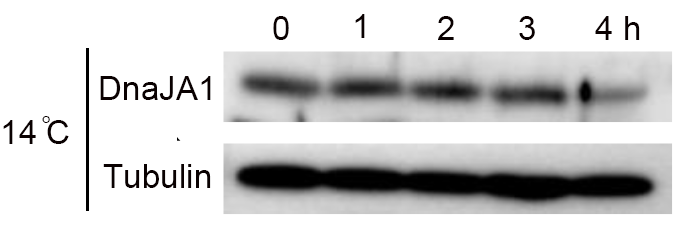

Supplement: FIGURE S2 — The protein levels of AccDnaJA1 under 14°C treatment. The target proteins were immunoblotted with anti-AccDnaJA1 and tubulin was used as a control. [file Image_2.TIF]
