# Supplementary material for: Environmental Stress Responses of DnaJA1, DnaJB12 and DnaJC8 in Apis cerana cerana
Source: Front Genet. 2018 Oct 8;9:445. doi: 10.3389/fgene.2018.00445 (PMC6186841; doi:10.3389/fgene.2018.00445)
Supplement: TABLE S1 — The collection times after treatment for the experimental groups. [file Table_1.doc]

**Table S1 The collection times after treatment for the experimental groups.**

| **Experiment stress conditions** | **Experiment stress conditions** | **Collection time posttreatment** |
| --- | --- | --- |
| Cold stress | 4 °C | 0.0, 1.0, 2.0, 3.0, 4.0 and 5.0 h |
|  | 14 °C | 0.0, 1.0, 2.0, 3.0, 4.0 and 5.0 h |
|  | 24 °C | 0.0, 1.0, 2.0, 3.0, 4.0 and 5.0 h |
| Pesticide stress | Lambda-cyhalothrin | 0.0, 0.5,1.0, 1.5, 2.0 and 3.5 h |
|  | Paraquat | 0.0, 1.0, 2.0, 4,0, 5.0 and 6.0 h |
|  | Emamectin benzoate | 0.0, 0.5, 1.0, 2.0, 3.0 and 4.0 h |
|  | Spirodiclofen | 0.0, 0.5, 1.5, 2.0, 3.0 and 3.5 h |
|  | Avermectin | 0.0, 1.0, 1.5, 2.0, 2.5 and 4.5 h |
| Heavy metal stress | CdCl_2_ | 0.0, 1.0, 2.0, 3.0, 4.0 and 5.0 h |
|  | HgCl_2_ | 0.0, 1.0, 2.0, 3.0, 4.0 and 5.0 h |
| Other stress | UV | 0.0, 1.0, 2.0, 3.0, 4.0 and 5.0 h |
