# Supplementary material for: Environmental Stress Responses of DnaJA1, DnaJB12 and DnaJC8 in Apis cerana cerana
Source: Front Genet. 2018 Oct 8;9:445. doi: 10.3389/fgene.2018.00445 (PMC6186841; doi:10.3389/fgene.2018.00445)
Supplement: TABLE S2 — Primer sequences used in this study. [file Table_2.docx]

Table S2 Primer sequences used in this study.

| Abbreviation | Primer sequence (5′–3′) | Description |
| --- | --- | --- |
| A1RTF | GTGGCAATGTATTCTCTTCA | qRT-PCR primer of *AccDnaJA1*, forward |
| A1RTR | CATCTTGTCCTCTGTGTTCT | qRT-PCR primer of *AccDnaJA1*, reverse |
| B12RTF | AAAGTGCTCAAGCTCATC | qRT-PCR primer of *AccDnaJB12*, forward |
| B12RTR | GAAATCCACCTCCAAAGA | qRT-PCR primer of *AccDnaJB12*, reverse |
| C8RTF | TGTCAATTCTTGTACATCCT | qRT-PCR primer of *AccDnaJC8*, forward |
| C8RTR | ACCTTCTTCTGTTTCTTCTT | qRT-PCR primer of *AccDnaJC8*, reverse |
| *β-actinRTR* | TTATATGCCAACACTGTCCTTT | Standard control qRT-PCR primer, forward |
| *β-actinRTF* | AGAATTGATCCACCAATCCA | Standard control qRT-PCR primer, reverse |
| A1RNAiF | TAATACGACTCACTATAGGGCGAATGGTAAAAGAAACTACATTTTATGATG | RNAi primer of *AccDnaJA1*, forward |
| A1RNAiR | TAATACGACTCACTATAGGGCGAAACCATGACATGTTGAACATTGC | RNAi primer of *AccDnaJA1*, reverse |
| B12RNAiF | TAATACGACTCACTATAGGGCGACTTCAAAGTGCTCAAGCTCA | RNAi primer of *AccDnaJB12*, forward |
| B12RNAiR | TAATACGACTCACTATAGGGCGACTTGCTTTCCACATCATTGT | RNAi primer of *AccDnaJB12*, reverse |
| C8RNAiF | TAATACGACTCACTATAGGGCGAATGGCTGCTACCAACTCGAATAT | RNAi primer of *AccDnaJC8*, forward |
| C8RNAiR | TAATACGACTCACTATAGGGCGAGTAACCTTCTTCTGTTTCTTCTTC | RNAi primer of *AccDnaJC8*, reverse |
