# Supplementary material for: Environmental Stress Responses of DnaJA1, DnaJB12 and DnaJC8 in Apis cerana cerana
Source: Front Genet. 2018 Oct 8;9:445. doi: 10.3389/fgene.2018.00445 (PMC6186841; doi:10.3389/fgene.2018.00445)
Supplement: TABLE S3 — PCR amplification procedures used in this study. [file Table_3.DOC]

Table S3 PCR amplification procedures used in this study.

| **Primer pair** | **Amplification conditions** |
| --- | --- |
| A1RNAiF/A1RNAiR | 10 min at 94 °C, 40 s at 94 °C, 40 s at 50 °C, 30 s at 72 °C for 30 cycles, 10 min at 72 °C |
| B12RNAiF∕B12RNAiR | 10 min at 94 °C, 40 s at 94 °C, 40 s at 49.5 °C, 30 s at 72 °C for 30 cycles, 10 min at 72 °C |
| C8RNAiF∕C8RNAiR | 10 min at 94 °C, 40 s at 94 °C, 40 s at 55.5 °C, 30 s at 72 °C for 30 cycles, 10 min at 72 °C |
